# Generalized tonic-clonic seizures as the initial symptom of late-onset Krabbe disease: a Case Report

**DOI:** 10.3389/fnbeh.2025.1564676

**Published:** 2025-11-06

**Authors:** Sifen Xie, Zuying Kuang, Mengqiu Pan, Kanghua Zhang, Jinlong Ye, Bo Li, Sheng Luo, Zhanhang Wang

**Affiliations:** 1 Department of Neurology, Guangdong Sanjiu Brain Hospital, Guangzhou, China; 2 Department of Neurology, Institute of Neuroscience, Key Laboratory of Neurogenetics and Channelopathies of Guangdong Province and the Ministry of Education of China, The Second Affiliated Hospital, Guangzhou Medical University, Guangzhou, China

**Keywords:** globoid cell leukodystrophy, epilepsy, *GALC* gene, adult-onset, cortical gray matter

## Abstract

Krabbe disease (KD), also known as globoid cell leukodystrophy, is a rare autosomal recessive neurodegenerative disorder caused by pathogenic variants in the *GALC* gene. While infantile-onset KD is prevalent globally, adult-onset KD is frequently presented in East Asian populations and typically manifests with progressive spastic paraparesis. We herein report a unique case of a 28-years-old male who initially presented with generalized tonic-clonic seizures, rather than the classic gait disturbance. Brain MRI revealed symmetrical white matter lesions and early cortical involvement. Genetic testing revealed compound heterozygous *GALC* variants (c.908C > T/p.Ser303Phe and c.136G > T/p.Asp46Tyr). Subsequent enzyme assays confirmed low galactocerebrosidase activity. This case broadens the clinical spectrum of adult-onset KD and highlights the importance of considering KD in the differential diagnosis of adult epilepsy with progressive neurological symptoms.

## Introduction

Krabbe disease (KD) is an autosomal recessive leukodystrophy caused by mutations in the *GALC* gene, which encodes galactocerebrosidase. Enzyme deficiency leads to the accumulation of psychosine and widespread demyelination in the central and peripheral nervous systems (Suzuki and Suzuki, 1970). KD is generally classified into four subtypes based on the age of onset: infantile, late-infantile, juvenile, and adult (Bascou et al., 2018; Madsen et al., 2018). The adult form typically emerges after the age of 16 (Bascou et al., 2018).

Although adult-onset KD commonly presents with chronic progressive gait disturbance, spastic paraparesis, and visual or swallowing difficulties, its clinical manifestations are highly variable (Debs et al., 2013; Malandrini et al., 2013). Epilepsy/seizures is a rare initial symptom, with only a few isolated case reports documenting myoclonic or other generalized seizures (Hwang et al., 2024; Wang et al., 2022). In this report, we reported a patient with adult-onset KD, but manifesting seizures as the initial symptom, rather than the more typical paraparesis.

## Case presentation

### Patient history

**FIGURE 1:**
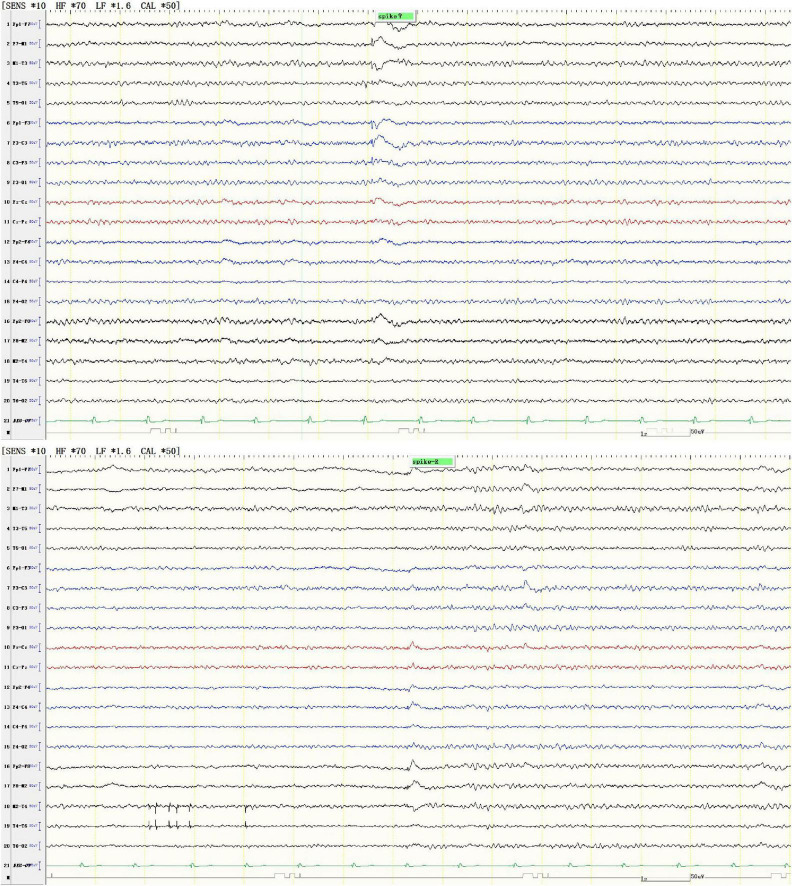
Representative EEG showed diffuse spike and/or spike-slow waves, predominantly in the left temporal lobe (upper panel) or the right temporal lobe (lower panel).

A 28-years-old male was admitted, who had a 12-years history of episodic loss of consciousness and a 2-years history of gait difficulty. At the age of 16 years and 7 months, he experienced his first episode of generalized tonic-clonic seizures (GTCS), manifesting sudden loss of consciousness, upward eye deviation, oral foaming, and generalized tonic-clonic limb contractions. Bilateral lower limb muscle atrophy is present. Proximal muscle strength in bilateral lower limbs is 4+/5, and distal muscle strength in bilateral lower limbs is 4/5. Bilateral knee and Achilles reflexes are hyperactive. Bilateral Babinski sign is positive (+). Pes cavus (high-arched feet) is noted. Other neurological examinations were normal. Electroencephalography (EEG) detected epileptiform discharges, characterized by diffuse spike and/or spike-slow wave with dominance in the left or the right temporal lobe (Figure 1). He was treated with valproic acid at a local hospital. However, this patient had poor compliance, often missed taking the medicine, stayed up late, and frequently played video games. His seizures persisted at a frequency of three to four episodes per month, which was often triggered by fatigue or irregular sleep patterns.

**FIGURE 2:**
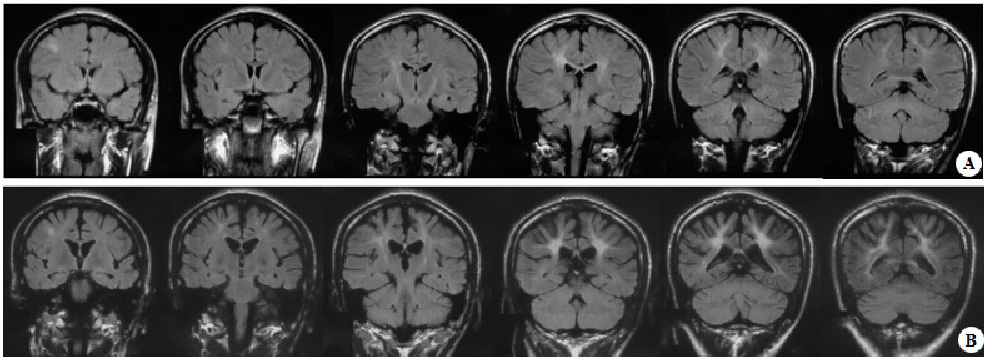
Representative brain MRI at the ages of 16 and 26 years (T2 FLAIR, coronal planes). **(A)** Brain MRI at the age of 16 detected symmetrical hyperintensities in the bilateral periventricular white matter and bilateral corticospinal tracts, as well as patchy hyperintensity in the right frontal subcortical region. **(B)** Brain MRI at the age of 26 detected symmetrical lesions in the bilateral corticospinal tracts, optic radiations, splenium of the corpus callosum, bilateral corona radiata, and the centrum semiovale (pre- and postcentral gyri) white matter, along with newly observed diffuse cerebral atrophy.

**FIGURE 3:**
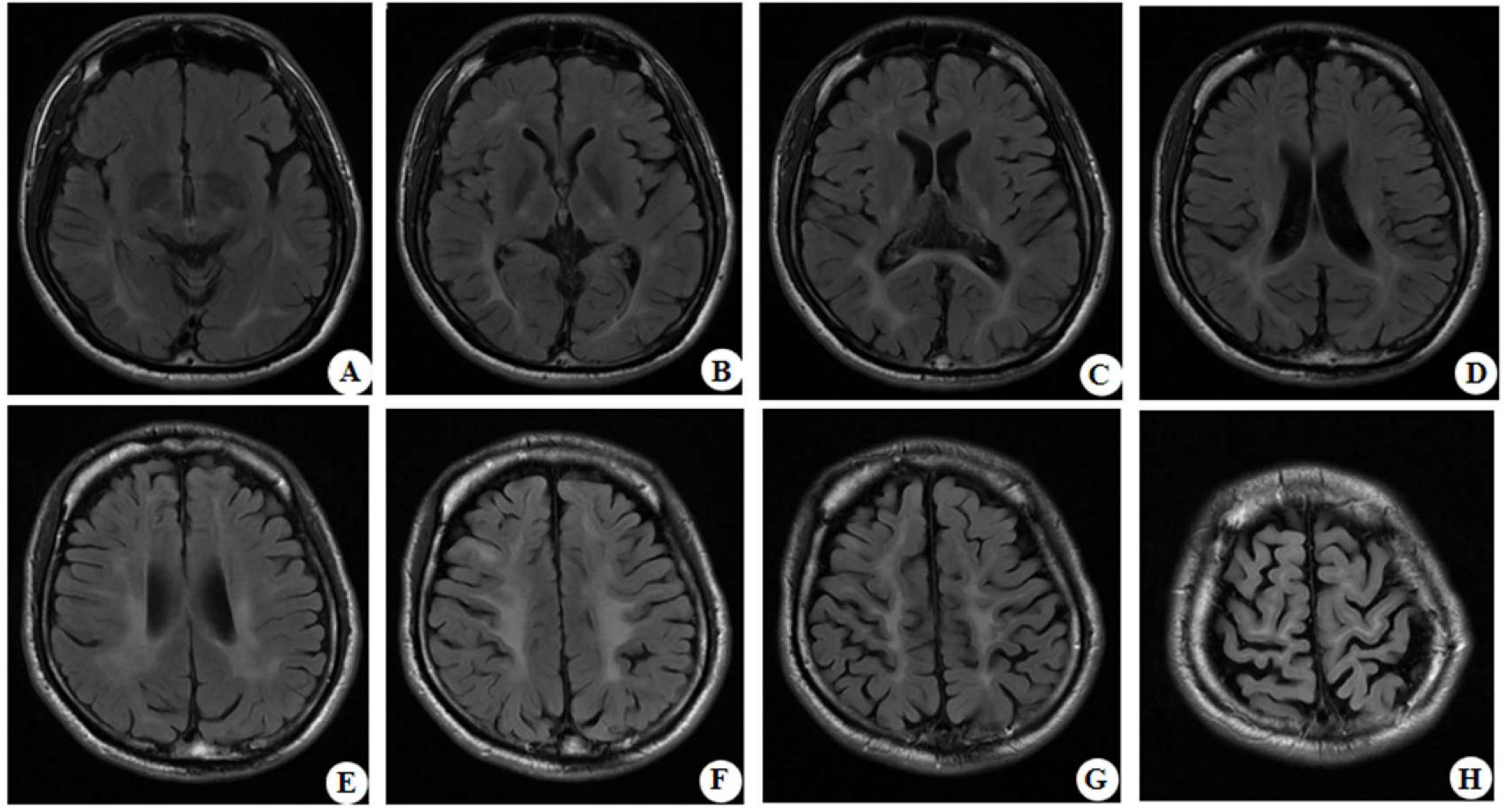
Representative brain MRI at the age of 28 years (T2 FLAIR, axial planes). Symmetrical abnormal signals are observed in the subcortical white matter of the bilateral frontal, temporal, parietal, and occipital lobes, the bilateral frontal and parietal cortex, the bilateral posterior limbs of the internal capsules, the bilateral corona radiata, the bilateral centrum semiovale, and the splenium of the corpus callosum.

**FIGURE 4:**
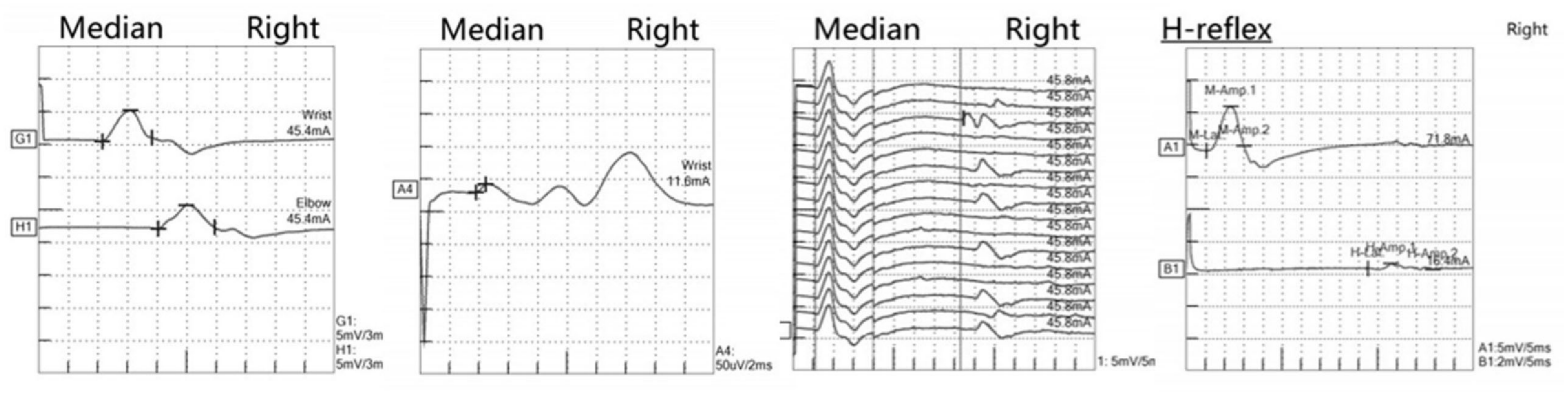
Neurophysiological examination revealed electrodiagnostic features of peripheral nerve and nerve root damage. The left two panels show median nerve motor conduction studies (upper) and sensory nerve conduction studies (lower), both exhibiting reduced amplitude responses and decreased conduction velocities, consistent with mixed axonal-demyelinating peripheral nerve damage. The middle panel displays median nerve F-wave recordings, revealing prolonged latencies and dispersed waveforms, indicative of pathological changes at the proximal nerve or root level. The rightmost panel presents tibial nerve H-reflex examination, demonstrating a decreased H/M ratio with asymmetrical responses, further supporting involvement at the nerve root level. The integrated electrophysiological findings, in correlation with clinical presentation, are characteristic of peripheral neuropathy with concomitant proximal nerve root involvement.

At the age of 16 years, his brain MRI showed bilateral high-signal abnormalities in the periventricular white matter and corticospinal tracts, along with a lesion in the right frontal subcortical region (Figure 2A). And the age of 26 detected symmetrical lesions in the bilateral corticospinal tracts, optic radiations, splenium of the corpus callosum, bilateral corona radiata, and the centrum semiovale (pre- and postcentral gyri) white matter, along with newly observed diffuse cerebral atrophy (Figure 2B). Subsequent MRIs conducted in 28 years old demonstrated progressive involvement of the frontal, parietal, temporal, and occipital lobes (Figures 3A–H). Physical examination revealed pes cavus (high-arched feet). While the patient denied marked sensory deficits, the neuroelectrophysiological examination revealed peripheral nerve damage, mainly demyelinating changes, and bilateral abnormal upper and lower limb sensory evoked potentials (Figure 4). So far, he has not exhibited significant visual decline. Ophthalmologic evaluation at Zhongshan Ophthalmic Center confirmed corrected visual acuity of 1.0 bilaterally, normal visual fields on perimetry, and unremarkable fundus examination, excluding optic nerve involvement. Cognitive assessment using the Mini-Mental State Examination (MMSE) yielded a score of 28/30 (adjusted for junior high education level), indicating preserved cognitive function. Laboratory test results were normal, including the amino acid and acylcarnitine spectrum analysis, content of very long chain fatty acids, adrenocorticotrophic hormone, cortisol, and creatine kinase.

### Genetic testing and enzyme assays

**FIGURE 5:**
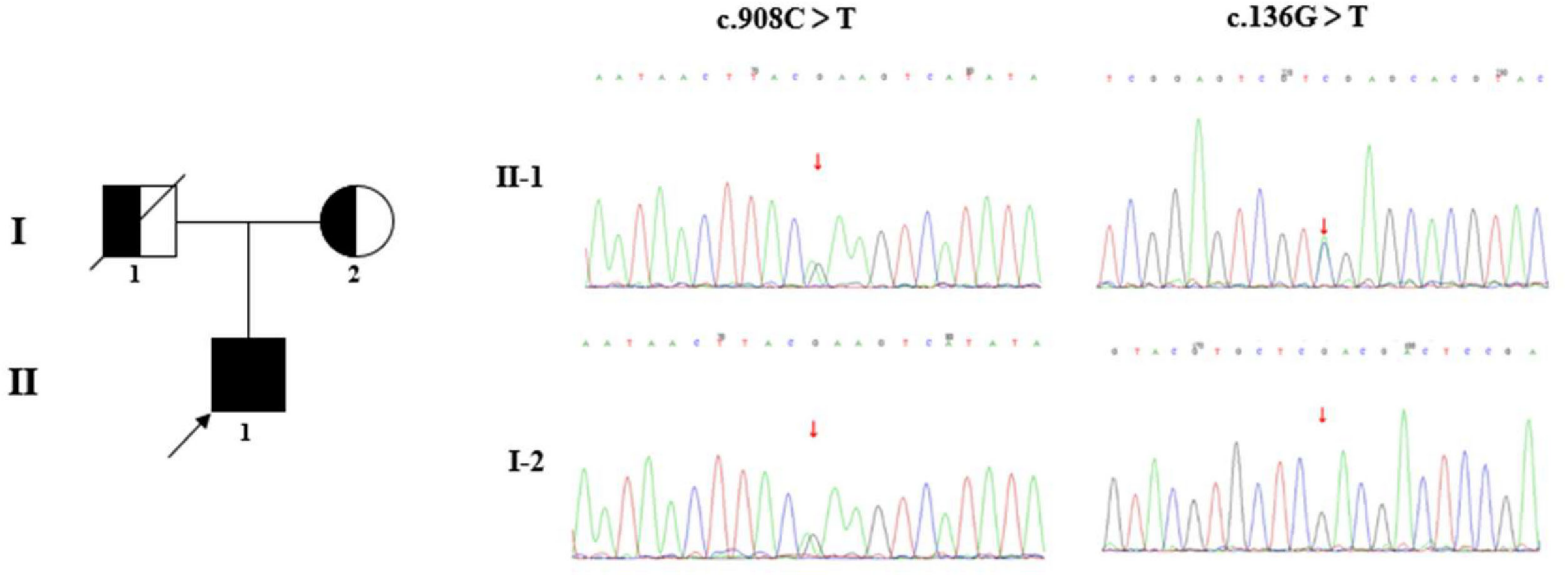
Family diagram and DNA sequencing chromatograms. The pedigree chart shows the patient’s father (I-1) and mother (I-2). The proband (II-1) is indicated by a black arrow. The c.908C > T/p.Ser303Phe was inherited from his mother, while the origin of the variant c.136G>T/p.Asp46Tyr was undetermined, due to the unavailable sample of his father (passed away). It is presumed that the variant c.136G>T/p.Asp46Tyr was inherited from his father.

Given this atypical presentation and the progressive white matter pathology, genetic testing was performed via the whole-exome sequencing method described in previous studies (He et al., 2024; Jin et al., 2023; Liu et al., 2024; Luo et al., 2023, 2025a,b; Yan et al., 2023; Yan et al., 2025a,b; Zhang et al., 2024). The patient is the only child from a non-consanguineous family in which no family members had similar neurological disorders, and the father passed away. The samples of the proband and his mother were then obtained to conduct whole-exon sequencing (Figure 5). Compound heterozygous mutations in the *GALC* gene (c.908C > T/p.Ser303Phe and c.136G > T/p.Asp46Tyr) were identified. The two variants were predicted to be damaging by multiple *in silico* tools (Table 1). According to the American College of Medical Genetics and Genomics guidelines, the two variants were both classified as likely pathogenic due to the same amino acid alterations as the variants previously identified as pathogenic (PS1), very low population frequency (PM2), pathogenic variants detected at the trans site (PM3), prediction as pathogenic by multiple *in silico* tools (PP3). We then conducted GALC enzyme analysis, which showed severely reduced galactocerebrosidase activity (∼2 nmol/17 h/mg; normal range 18∼75 nmol/17 h/mg). Taken together, the patient was confirmed to be diagnosed with Krabbe disease.

**TABLE 1:** Predicting the damaging effects of identified *GALC* variants by 18 *in silico* tools.

Algorithm	Prediction/score	
	c.908C > T/p.Ser303Phe	c.136G > T/p.Asp46Tyr
1. SIFT	Damaging/0.003	Damaging/0.013
2. Polyphen-2_HDIV	Possibly damaging/1.0	Possibly damaging/1.0
3. Polyphen-2_HVAR	Possibly damaging/0.998	Possibly damaging/0.991
4. LRT	Deleterious/0.000	Deleterious/0.000
5. MutationTaster	Disease-causing/1	Disease-causing/1
6. ClinPred	Pathogenic/0.8675	Pathogenic/0.7749
7. MutationAssessor	Medium/2.79	Medium/2.79
8. FATHMM	Damaging/−5.62	Damaging/−3.66
9. PROVEAN	Damaging/−4.56	Damaging/−3.91
10. VEST3	Damaging/0.886	Damaging/0.708
11. MetaSVM	Damaging/1.074	Damaging/0.914
12. MetaLR	Damaging/0.978	Damaging/0.902
13. M-CAP	Damaging/0.615	Damaging/0.641
14. CADD	Damaging/34	Damaging/34
15. DANN	Damaging/0.998	Damaging/0.995
16. FATHMM_MKL	Damaging/0.982	Damaging/0.800
17. Eigen	Damaging0.816	Damaging0.484
18. GenoCanyon	Damaging/1.000	Damaging/1.000
19. fitCons	Tolerable/0.693	Tolerable/0.442
20. ReVe	Damaging/0.91800451	Damaging/0.84636199
21. phyloP	Conserved/9.878	Conserved/5.143
22. phastCons	Conserved/1.000	Conserved/1.000
23. SiPhy	Conserved/16.727	Conserved/14.347
24. REVEL	Conserved/0.957	Conserved/0.841
25. GERP++	Conserved/4.68	Conserved/3.21

CADD, combined annotation dependent depletion; FATHMM, functional analysis through hidden Markov models; fitCons, the fitness consequences of functional annotation; GERP, genomic evolutionary rate profiling; LRT, likelihood ratio test; M-CAP, Mendelian clinically applicable pathogenicity score; phastCons, conservation scoring and identification of conserved elements; phyloP, computation of *p*-values for conservation or acceleration, either lineage-specific or across all branches; Polyphen-2_HVAR, HumVar-trained PolyPhen-2; PROVEAN, protein variation effect analyzer; REVEL, rare exome variant ensemble learner; ReVe, a combination of the predictions of REVEL and VEST4 (variant effect scoring tool 4.0). Detailed predicted scores were obtained from the VarCards database (http://www.genemed.tech/varcards/index.php).

### Treatment and follow-up

Extended-release sodium valproate (Depakine^®^; 0.5 g bid) was maintained for the treatment of seizures. Supportive therapies included coenzyme Q10, idebenone, and B-group vitamins, which are standard neurotrophic agents used in the treatment of leukodystrophies. Despite the reduction in seizure frequency, the patient’s gait problems worsened, necessitating the use of a walking aid. At 6-months follow-up, no new seizures were recorded, but motor deficits persisted. The improvement in seizure control was likely due to improved compliance rather than solely the effect of supportive therapy.

### Review of previously reported cases with variants p.Asp46Tyr or p.Ser303Phe

The two *GALC* variants identified in this case were previously reported in several cases. We thus reviewed those cases to explore possible genotype-phenotype correlation. To our knowledge, a total of four cases have been identified, including three cases with variant p.Asp46Tyr and one case with variant p.Ser303Phe (Supplementary Table 1). The case with homozygous variants p.Asp46Tyr exhibited adult-onset Krabbe Disease with left upper limb wasting and weakness as the initial symptom (Wu et al., 2023), while the other three cases harboring compound heterozygous variants exhibited earlier-onset Krabbe Disease (Dong et al., 2020; Lin et al., 2020; Zhuang et al., 2019). Seizures were only documented in a case, but without detailed information (Lin et al., 2020). Notably, the *GALC* enzymatic activity of cases with seizures was relatively higher than those of cases without seizures, potentially suggesting a possible association between the damage of enzymatic activity and phenotypic severity.

## Discussion

Adult-onset KD comprises only about 5% of all KD cases, yet it is relatively more common in East Asian populations (Bascou et al., 2018; Madsen et al., 2018). Most adult patients present with progressive spastic paraparesis, peripheral neuropathy, and occasional visual impairment (Debs et al., 2013; Malandrini et al., 2013). This case is distinctive in that GTCS was the initial clinical manifestation, a rarity also noted in only one previously reported patient of adult-onset KD with progressive myoclonus epilepsy (Wang et al., 2022). This previously reported patient had a homozygous GALC variant c.1901T > C/p.Leu634Ser and a reduced GALC enzyme activity of ∼3.3 nmol/17 h/mg.

Regarding genetics, known hot-spot mutations in *GALC* vary by ethnicity. In European patients, large deletions and specific point mutations, such as p.Thr529Met, are prevalent (Madsen et al., 2019). In China, point mutations p.Leu634Ser are often observed in late-onset KD (Wang et al., 2022; International Journal of Neurology and Neurosurgery, 2024). In this study, the patient harbored two *GALC* variants, p.Ser303Phe and p.Asp46Tyr. While the two variants were sporadically reported in single patients (Wenger et al., 1997; Zhuang et al., 2019), the compound heterozygous variants constituted by the two variants were novel. Further enzyme assays indicated the low activity of galactocerebrosidase, validating the pathogenicity of variants. Then, the precision diagnosis of KD was achieved, explaining his unusual but extended disease course over more than a decade. To date, more than 325 pathogenic variants in *GALC* have been identified (obtained from the HGMD database, April 2024), among which the majority were of missense. In genetics, missense variants were the most common variant type, with large uncertainty significance of pathogenicity. The identification of novel compound heterozygous variants in the presented study, which are composed of previously reported pathogenic variants, expanded the *GALC* mutation spectrum and aided in interpreting the pathogenicity of missense variants, particularly for guiding clinical genetic testing in autosomal recessive KD. When identifying a single previously reported pathogenic missense variant in genes of an autosomal recessive inheritance pattern, it should be given attention to identifying variants in another chromosome (*in trans* variants) in the families/cases to achieve early genetic diagnosis, genetic counseling, and/or prenatal diagnosis. A multidisciplinary approach involving neurologists, geneticists, and specialists in metabolic disorders is crucial for timely and accurate diagnosis.

Neuroimaging of KD typically shows symmetrical lesions in the periventricular white matter, corticospinal tracts, and optic radiations (Cousyn et al., 2019; Muthusamy et al., 2019). In the patient of this study, MRI revealed not only characteristic white matter abnormalities but also initial right frontal subcortical signal abnormalities, progressing to involve bilateral fronto-parietal cortex and cortical atrophy as the disease developed. Notably, Ketata et al. described parieto-occipital lesions in KD, linking to psychosine-mediated demyelination and neuroinflammation (Ketata and Ellouz, 2024). The fronto-subcortical findings of this study may share similar pathogenesis, reflecting regional vulnerability to psychosine toxicity. It is possible that KD had a broader spectrum of radiological patterns, underscoring the need for further studies to clarify its pathophysiology, clinical significance, and potential prognostic implications.

The cortical or subcortical gray matter changes observed in the presented patients might predispose to epileptogenesis, which may be the possible explanation of the observed initial symptom of GTCS. This finding expands the imaging spectrum of KD, suggesting that clinicians should consider the differential diagnosis of KD for adult epilepsy patients with frontal lobe abnormalities. Notably, the patient later developed spastic paraparesis, high-arched feet, and peripheral nerve involvement, paralleling the typical adult-onset KD phenotypes. The unique compound heterozygous variants in this patient may be associated with the atypical clinical course. As the disease progresses, the patient gradually shows typical adult-onset KD symptoms, suggesting that despite the difference in the genetic background, there are still certain commonalities in the disease development process, although the initial stage and the progression rate may vary. However, this hypothesis requires further verification through functional studies and the accumulation of more cases.

Early recognition of later-onset KD presenting with epilepsy remains challenging, frequently leading to delayed diagnosis. When adult patients present with unexplained seizures and subtle neurological signs, such as pes cavus, an inherited leukodystrophy like KD should be considered. Genetic testing, enzymatic assays, and laboratory tests including amino acid and acylcarnitine spectrum analysis, measurement of very long chain fatty acids, adrenocorticotrophic hormone, and cortisol, are crucial for distinguishing KD from other leukodystrophies or demyelinating disorders (e.g., adrenoleukodystrophy). Hematopoietic stem cell transplantation (HSCT) may benefit some KD patients if performed early, though efficacy in adult-onset disease remains uncertain (International Journal of Neurology and Neurosurgery, 2024). Research on the underlying pathophysiology of KD and the development of novel therapeutic strategies, including targeted gene therapies and disease-modifying drugs, would offer hope for improved outcomes in the future.

## Conclusion

We report a patient with adult-onset KD presenting initially with epilepsy, likely reflecting early cortical involvement. Although adult-onset KD often presents with progressive paraparesis, clinicians should remain vigilant to the possibility of KD in adults with new-onset seizures. Prompt neuroimaging, genetic screening, and enzyme assays may improve patient outcomes by facilitating early intervention. This study broadens the clinical spectrum of adult-onset KD and underscores the importance of considering KD in the differential diagnosis of adult epilepsy with progressive neurological symptoms.

## Data Availability

The data presented in the study has been deposited in the GenBank repository, accession number PX120100 and PX120101.
