# Correction: Generalized tonic-clonic seizures as the initial symptom of late-onset Krabbe disease: a Case Report

**DOI:** 10.3389/fnbeh.2025.1741000

**Published:** 2025-12-01

**Authors:** Sifen Xie, Zuying Kuang, Mengqiu Pan, Kanghua Zhang, Jinlong Ye, Bo Li, Sheng Luo, Zhanhang Wang

**Affiliations:** 1 Department of Neurology, Guangdong Sanjiu Brain Hospital, Guangzhou, China; 2 Department of Neurology, Institute of Neuroscience, Key Laboratory of Neurogenetics and Channelopathies of Guangdong Province and the Ministry of Education of China, The Second Affiliated Hospital, Guangzhou Medical University, Guangzhou, China

**Keywords:** globoid cell leukodystrophy, epilepsy, *GALC* gene, adult-onset, cortical gray matter

In the published article, the wrong image for Figure 2 was erroneously used and was duplicated from the image of Figure 3. The corrected Figure 2 appears below.

**Figure 2:**
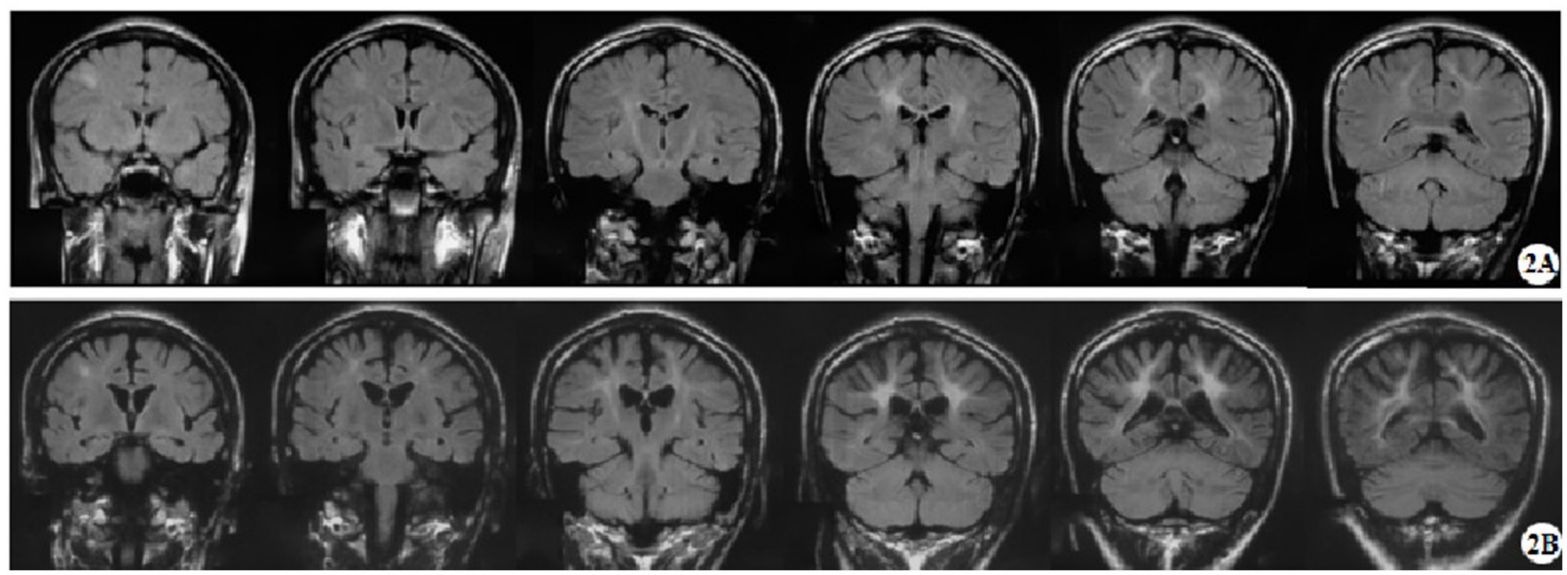
Representative brain MRI at the ages of 16 and 26 years (T2 FLAIR, coronal planes). **(A)** Brain MRI at the age of 16 detected symmetrical hyperintensities in the bilateral periventricular white matter and bilateral corticospinal tracts, as well as patchy hyperintensity in the right frontal subcortical region. **(B)** Brain MRI at the age of 26 detected symmetrical lesions in the bilateral corticospinal tracts, optic radiations, splenium of the corpus callosum, bilateral corona radiata, and the centrum semiovale (pre- and postcentral gyri) white matter, along with newly observed diffuse cerebral atrophy.

The original version of this article has been updated.

