# Supplementary material for: Generalized tonic-clonic seizures as the initial symptom of late-onset Krabbe disease: a Case Report
Source: Front Behav Neurosci. 2025 Nov 6;19:1564676. doi: 10.3389/fnbeh.2025.1564676 (PMC12631614; doi:10.3389/fnbeh.2025.1564676)
Supplement: Supplementary file 1 [file Table_1.docx]

**Table S1. Previously reported cases with *GALC* variants c.136G>T/p.Asp46Tyr or c.136G>T/p.Asp46Tyr**

| **Cases** | Case 1 | Case 2-1 | Case 2-1 | Case 3 | Case 4 | Case 5 |
| --- | --- | --- | --- | --- | --- | --- |
| **Variants 1** | c.136G>T/p.Asp46Tyr | c.136G>T/p.Asp46Tyr | | c.136G>T/p.Asp46Tyr | c.136G>T/p.Asp46Tyr | c.908C>T/p.Ser303Phe |
| **Variants 2** | c.908C>T/p.Ser303Phe | c.865G > C: p. G289R | | c.136G>T/p.Asp46Tyr | c.1586C>T/p.Thr529Met | c.466 T > C/p.Trp156Arg |
| **Diagnose** | Adult-onset Krabbe Disease | Juvenile-onset Krabbe Disease | | Adult-onset Krabbe Disease | Developmental disorder | Krabbe Disease |
| **Enzymatic activity** | 2 nmol/17h/mg | 3.9 nmol/mg/17 h | 4.4 nmol/mg/17 h | NA | NA | 0.2 nmol/mg/17 h |
| **Age at onset** | 16 years sand 7 months | 5 years | 4 years | 38 years | NA | NA |
| **Initial symptom** | generalized tonic-clonic seizures | mild gait difficulties, unstable ambulation | Development regression | Left upper limb wasting and weakness | NA | Development regression |
| **Other symtoms** | Walking impairment | Vision loss | Walking impairment | Pyramidal signs | NA | Development regression and generalized seizures |
| **Epilepsy** | Yes | No | No | No | NA | NA |
| **Cognitive** | Normal | Normal | Regression | NA | NA | NA |
| **MRI** | Bilateral high-signal abnormalities in the periventricular white matter and corticospinal tracts, along with a lesion in the right frontal subcortical region | high-intensity signal in the left central gyrus cortex | NA | NA | NA | Hydrocephalus, Demyelination |
| **EEG** | diffuse spike and/or spike-slow wave with dominance in the left or the right temporal lobe | NA | NA | NA | NA | NA |
| **Reference** | This study | [1] | | [2] | [3] | [4] |

Reference:

1. Zhuang S, Kong L, Li C, Chen L, Zhang T: **GALC mutations in Chinese patients with late-onset Krabbe disease: a c ase report**. *BMC neurology*, **19**(1):122.

2. Wu C, Wang M, Wang X, Li W, Li S, Chen B, Niu S, Tai H, Pan H, Zhang Z: **The genetic and phenotypic spectra of adult genetic leukoencephalopath ies in a cohort of 309 patients**. *Brain : a journal of neurology*, **146**(6):2364-2376.

3. Dong X, Liu B, Yang L, Wang H, Wu B, Liu R, Chen H, Chen X, Yu S, Chen B *et al*: **Clinical exome sequencing as the first-tier test for diagnosing develo pmental disorders covering both CNV and SNV: a Chinese cohort**. *Journal of medical genetics*, **57**(8):558-566.

4. Lin L, Zhang Y, Pan H, Wang J, Qi Y, Ma Y: **Clinical and genetic characteristics and prenatal diagnosis of patient s presented GDD/ID with rare monogenic causes**. *Orphanet Journal of Rare Diseases*, **15**(1).
